# Annotation of pituitary neuroendocrine tumors with genome-wide expression analysis

**DOI:** 10.1186/s40478-021-01284-6

**Published:** 2021-11-10

**Authors:** Abdellah Tebani, Jelena Jotanovic, Neda Hekmati, Åsa Sivertsson, Olafur Gudjonsson, Britt Edén Engström, Johan Wikström, Mathias Uhlèn, Olivera Casar-Borota, Fredrik Pontén

**Affiliations:** 1 Science for Life Laboratory, Department of Protein Science, KTH-Royal Institute of Technology, Stockholm, Sweden; 2 Department of Metabolic Biochemistry, UNIROUEN, INSERM U1245, CHU Rouen, Normandie University, 76000 Rouen, France; 3 Department of Immunology, Genetics and Pathology, Uppsala University, Uppsala, Sweden; 4 Department of Clinical Pathology, Uppsala University Hospital, Uppsala, Sweden; 5 Department of Neuroscience, Uppsala University, Uppsala, Sweden; 6 Department of Medical Sciences, Endocrinology and Mineral Metabolism, Uppsala University, Uppsala, Sweden; 7 Department of Surgical Sciences, Neuroradiology, Uppsala University, Uppsala, Sweden

**Keywords:** PitNET, Transcriptomics, RNA-seq, Pituitary adenoma, Pathology, Omics

## Abstract

Pituitary neuroendocrine tumors (PitNETs) are common, generally benign tumors with complex clinical characteristics related to hormone hypersecretion and/or growing sellar tumor mass. PitNETs can be classified based on the expression pattern of anterior pituitary hormones and three main transcriptions factors (TF), SF1, PIT1 and TPIT that regulate differentiation of adenohypophysial cells. Here, we have extended this classification based on the global transcriptomics landscape using tumor tissue from a well-defined cohort comprising 51 PitNETs of different clinical and histological types. The molecular profiles were compared with current classification schemes based on immunohistochemistry. Our results identified three main clusters of PitNETs that were aligned with the main pituitary TFs expression patterns. Our analyses enabled further identification of specific genes and expression patterns, including both known and unknown genes, that could distinguish the three different classes of PitNETs. We conclude that the current classification of PitNETs based on the expression of SF1, PIT1 and TPIT reflects three distinct subtypes of PitNETs with different underlying biology and partly independent from the expression of corresponding hormones. The transcriptomic analysis reveals several potentially targetable tumor-driving genes with previously unknown role in pituitary tumorigenesis.

**Supplementary Information:**

The online version contains supplementary material available at 10.1186/s40478-021-01284-6.

## Introduction

Pituitary neuroendocrine tumors, traditionally designated as pituitary adenomas, represent a biologically heterogenous group of neoplasms of adenohypophysial cell origin [2, 27]. Although well-characterized clinically and histologically, the global molecular landscape of PitNETs has been studied in only a few studies [35, 42, 47] and is, thus, still poorly understood. To enable a deeper understanding of PitNET biology and to facilitate the development of new diagnostic and therapeutic approaches, we have here applied genome-wide mRNA expression analysis to identify PitNET fingerprints. The discovery and validation of new proteins involved in growth and development of PitNETs is important to improve the classification of different forms of PitNET tumors and to revise controversial tumor categories. The data derived from our transcriptomics analyses provides a verification of the current PitNET classification and presents starting points for further analysis of several new proteins implicated in the field of PitNETs.

Current WHO classification of the PitNETs is based on the pituitary cell lineages [26, 27]. The pituitary cell lineages are defined by expression of anterior pituitary hormones and pituitary specific transcription factors (TFs): steroidogenic factor-1 (SF1), encoded by the *NR5A1* gene, that regulates differentiation of gonadotroph cells producing folliculostimulating (FSH) and luteinizing (LH) hormone [1, 56], pituitary transcription factor 1 (PIT1), encoded by the *POU1F1* gene, that determines the development of growth hormone (GH), prolactin (PRL) and thyroid stimulating hormone (TSH) producing cells [3, 23], and the T-box transcription factor TPIT, encoded by the *TBX19* gene, responsible for the development of corticotroph cells that produce adrenocorticotroph hormone (ACTH), encoded by the *POMC* gene [19, 37]. The commonly used names for these pituitary transcription factors (SF1, PIT1 and TPIT) and corresponding hormones (FSH, LH, GH, PRL, TSH and ACTH) will be used throughout the manuscript also for respective genes and gene transcripts.

Principally, the vast majority of PitNETs can be classified in one of the three categories: SF1 positive gonadotroph tumors, PIT1 positive somatotroph, lactotroph and thyrotroph tumors, or combinations of these, and TPIT positive corticotroph tumors [30]. In uncommon cases of true plurihormonal tumors, tumor cells belonging to at least two pituitary cell lineages are spread within the tumor [26]. Rare double and triple PitNETs consist of well-defined tumor components belonging to two or three different cell lineages [26]. Tumors immunonegative for adenohypophysial hormones and TFs have been classified as tumors of undetermined cell lineage or “null cell adenomas” [26]. Complementary use of antibodies towards anterior pituitary hormones and TFs has reduced the proportion of so called “null cell adenomas” to such a low percentage [36, 43] that their existence has been questioned [30]. PitNET of any histological type may cause symptoms due to hormone hypersecretion, such as acromegaly related to GH hypersecretion, Cushing disease related to ACTH hypersecretion, symptoms related to hyperprolactinaemia, TSH or, very rarely, FSH/LH hypersecretion, or behave as a silent tumor, without laboratory or clinical signs of hormone hypersecretion. Silent PitNETs represent approximately 50% of all PitNETs and cause symptoms related to growing sellar tumor mass including visual disturbances and anterior pituitary gland failure. A majority of silent PitNETs are of gonadotroph origin (> 80%), followed by corticotroph tumors (> 15%), and rarely other types [30]. Mechanisms behind silencing of PitNETs are largely unknown.

Immunohistochemistry with antibodies towards pituitary TFs has not yet been routinely implemented at many diagnostic laboratories, thus, pituitary cell-lineage based classification of PitNETs has not yet been validated in the clinical settings, except in one recent study [21]. Studies exploring how gene expression in PitNETs reflect the current classification in well-characterized tumor cohorts are limited [35, 47]. There are sparse data on the differential gene expression between clinically functioning (F-PitNET) and non-functioning PitNETs (NF-PitNET) [12]. More knowledge on gene expression in PitNETs would contribute to a better understanding of the biological plasticity of these tumors and help in the validation of the current WHO classification of pituitary tumors to further improve this classification, particularly regarding the controversial categories such as “null cell adenomas”.

Sequencing of mRNA from tissue has proven to be a powerful technology to reveal fingerprints of both normal and tumor tissues. Transcriptomics analyses allow for broad detection and deep characterization of gene expression signatures that correspond to different tissue and tumor types defined by morphologic assessment of the cells included in a composite tissue. Within large scale platforms such as the Human Protein Atlas (www.proteinatlas.org), efforts have been successful in creating transcriptomic profiles of human organs and tissues, to identify tissue type specific proteins and to distinguish the relation between different types of tissues [50]. Due to heterogeneity, gene expression profiling has, at large, yielded less clear and convincing data to establish tumor type specific fingerprints. Nevertheless, PitNETs represent a group of tumors with well-defined subgroups. As such, they present an attractive set of tumors for transcriptomic profiling with the aim to validate the current tumor classification based on immunohistochemistry and expression patterns of pituitary transcription factors and hormones.

In this study, we have performed genome-wide mRNA sequencing on 51 tumors from a well-characterized cohort of PitNETs that have been classified according to clinical endocrine symptoms and the current 2017 WHO classification of the pituitary tumors [26]. Our results show that the overall gene expression pattern in PitNETs is aligned with the current PitNET classification. We provide further evidence supporting need for revision of the category “null cell adenoma”. Moreover, we have identified transcripts that distinguish PitNETs driven by SF1, PIT1 and TPIT, some of them corresponding to the novel proteins with potential for diagnostics and pharmacological therapies.

## Material and methods

### Study cohort and tissue samples

Fifty-one tumor tissue samples were collected after transsphenoidal surgeries for pituitary tumors performed at the Uppsala University Hospital. Immunohistochemistry-based classification was performed as part of the clinical diagnostic procedure using standardized routine immunohistochemistry and the results are summarized in Table 1 with detailed case information in Additional file 2: Table S1. All tumors were classified based on the immunohistochemical expression of anterior pituitary hormones and pituitary specific transcription factors according to the WHO 2017 classification of the tumors of endocrine organs [26]. Fourteen tumors expressed SF1 and were all clinically non-functioning. Twenty tumors expressed PIT1, two were non-functioning whereas 18 were functioning. A single functioning FSH-oma paradoxically expressed PIT1. Thirteen tumors expressed TPIT, three caused Cushing’s disease, whereas remaining 10 were silent corticotroph tumors. One patient had a clinically non-functioning double gonadotroph plus lactotroph tumor and one patient had a triple PitNET with tumor components belonging to all three cell lineages. Only two tumors were immunonegative for all anterior pituitary hormones and pituitary specific transcription factors and were consequently classified as “null cell tumors”. The project has been approved by the Swedish Ethical Review Authority, Dnr 2018/053.

**Table 1:** Immunohistochemical and clinical features of the PitNETs

TF	n	Functioning status	n	IHC subtype			Clinical classification		Comment
SF1	14	NF	14	Gonadotroph			NF-PitNET		
PIT1	20	NF	2	Somatotroph		1	NF-PitNET		
				only Pit-1 positive		1			
		F	18	Somatotroph		10	Acromegaly		Two SG were mixed PitNET-gangliocytomas
				DG	4				
				SG	5				
				NG	1				
				Lactotroph		4	HyperPRL		
				Thyrotroph		1	HyperTSH		
				Plurihormonal GH + PRL + TSH		2	Acromegaly + HyperTSH	1	
							HyperPRL	1	
				Gonadotroph		1	HyperFSH		Paradoxical PIT1expression and not SF1
TPIT	13	NF	10	Corticotroph			NF-PitNET		One Crooke cell tumor
		F	3	Corticotroph			Cushing		
Double PitNET	1	NF	1	Gonadotroph + lactotroph			NF-PitNET		
Triple Pit-NET	1	F	1	GH + PRL + ACTH			Acromegaly		
Null cell	2	NF	2				NF-PitNET		
In total	51								

### Radiological evaluation

Pituitary magnetic resonance imaging (MRI) studies of the patients were reviewed regarding tumor volume and invasiveness. These assessments were performed on the last examination before first surgery. MRI protocols differed between the hospitals where the patients were primarily examined but included typically coronal and sagittal T1 weighted images at 2–3 mm slice thickness before and after contrast medium administration, and coronal or sagittal 2–3 mm thick T2 weighted images. Tumor size in three orthogonal directions (height, width, and depth) was measured on T1 weighted images after contrast medium administration. Tumor volume was calculated according to the expression for an ellipsoid: volume = (width × height × depth)/2. Degree of (parasellar) invasiveness was evaluated using the modified Knosp classification [33], into grades 0 to 4, where grades 3A, 3B, and 4 were regarded as signs of invasion.

### Immunohistochemistry

Selected cases were immunohistochemically stained with antibodies detecting IGSF1, IDH1, FKBP10, IKBIP and ACSL1 to validate results from the transcriptomics analyses and to explore corresponding protein expression patterns. An overview of the antibodies and immunohistochemical protocols used for PitNET classification and for examples of partly novel pituitary proteins are displayed in Additional file 2: Table S2.

### Transcriptome profiling (RNA-seq)

#### RNA extraction, library preparation and sequencing

All 51 tumors were sectioned and stained with hematoxylin–eosin to ascertain presence of representative tumor tissue in the frozen specimens used for RNA extraction. Three 10 µm thick sections from each frozen tissue block were collected and total RNA was extracted using the RNeasy Mini Kit (Qiagen, Hilden, Germany). The RNA samples were analyzed using automated electrophoresis system Experion (Bio-Rad Laboratories, Hercules, CA, USA) with the standard-sensitivity RNA chip or an Agilent 2100 Bioanalyzer system (Agilent Biotechnologies, Palo Alto, USA) with the RNA 6000 Nano LabChip Kit. High-quality total RNA with Integrity Number ≥ 7.5 was then used to prepare mRNA libraries for RNA sequencing using the llumina TruSeq Stranded mRNA kit. The libraries were sequenced on the Illumina NovaSeq 6000 instrument with 2 × 151 setup using 'NovaSeqXp' workflow in 'S4' mode flowcell.

#### Transcriptomics data pre-processing

Two main metrics have been used to assess the quality of RNAseq data: percentage of reads with quality score higher than 30 (Q-score ≥ 30), as provided by the sequencing pipeline, and the number of reads. The cut-off for the percentage of reads was set to 75% and the cut-off for the number of reads was set at 10 million reads. All samples have passed these QC limits. To obtain quantification scores for all human genes and transcripts across all samples, transcript expression levels were calculated as transcript per million (TPM) by mapping processed reads to the human reference genome GRCh37/hg19 ref and with gene models based on Ensembl (v92) using Kallisto (v.0.43.1) [6]. Next, the gene expression levels were calculated by summing up all the TPM values of all alternatively spliced protein coding transcripts of the corresponding gene for a total number of 19,670 protein-coding genes. The average TPM values are used to estimate the gene expression level. All TPM values were TMM normalized [40] between all the samples. Expression level cut-off is set at 1 TPM. A total number of 9677 genes are expressed at 1 TPM or higher in all samples. The full TPM data matrix is shown in Additional file 2: Table S3.

### Data analysis and visualization

Differential expression analysis was conducted by using mRNA raw counts. The DESeq2 R package [28] was used for differential analysis. Genes with false discovery rate (adjusted *p *value with “Benjamini–Hochberg” method [4]) less than 0.05 and absolute fold change higher than 2 were considered as differentially expressed genes. Data analysis and visualization was performed using on R (version 4.0.0) [48]. Clustering in heatmaps and dendrograms based on Spearman correlation were created by first calculating a correlation matrix of Spearman’s ρ [45] between all samples. The correlation was converted to a distance metric (1 − ρ) and was clustered using unsupervised top-down hierarchical clustering. Dendrograms showing gene expression in heatmaps have been clustered using the Ward2 algorithm an implementation of Ward’s minimum variance method [34] implemented as “Ward.D2” in the hclust function in the R package stats. Principal Component Analysis has been performed on log transformed values (log(TPM + 1)) using the R package pcaMethods [46].

## Results

### Transcriptome profiling

**Fig. 1:**
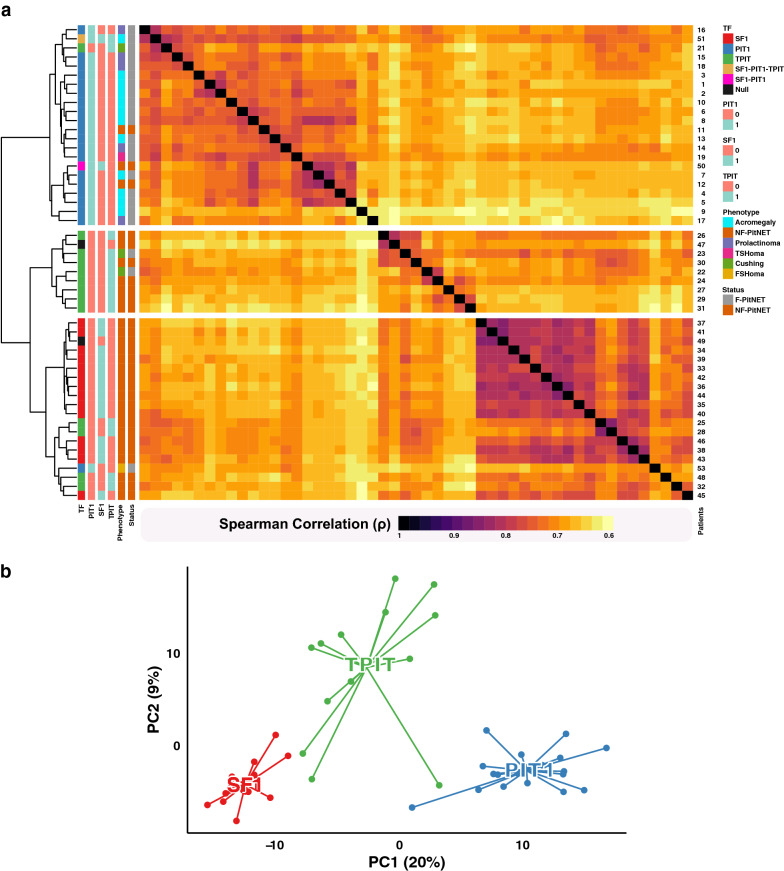
Overview of the expression profiles of the protein-coding genes in the human PitNETs. **a** A heatmap showing the pairwise Spearman correlation between the global gene expression profiles for the analyzed 51 pituitary tumor tissues. (F-PitNET: Functioning Pituitary Neuroendocrine Tumor, NF-PitNET: Non-Functioning Pituitary Neuroendocrine Tumor). **b** Principal component analysis showing the relationship between all the analyzed tumors and their respective transcription factor (TF) classes

The fraction of tumor cells in Hematoxylin–Eosin stained cryosections corresponding to the isolated RNA for sequencing was 80–90% for all tumors except four cases having 70% and one case with 50% tumor cells. The transcriptomics data was obtained from RNA sequencing of fresh frozen tissue and normalized mRNA levels were determined for each sample, calculated as transcript per million (TPM) values. In total 51 PitNETs were analyzed and with a cut-off value of 1 TPM, 16,803 genes were expressed in at least one PitNET sample and 9677 genes (49% of all putative protein coding genes) were found to be expressed in all 51 analyzed tumors. The global expression profiles of the 51 tumors were compared using hierarchical clustering, including a correlation heatmap (Fig. 1a). The results reveal three main clusters corresponding well to the expected categories of tumors based on the three main TFs (SF1, TPIT and PIT1), with the PIT1 cluster separated from the more closely related SF1 and TPIT clusters (Fig. 1a). Within the three main clusters there are six tumors that do not cluster as would be expected using the current immunohistochemistry-based classification. The SF1 cluster contains four non-functioning TPIT tumors and one FSH-producing PIT1 positive tumor. In the PIT1 cluster there was one functioning TPIT tumor. The two tumors classified as expressing > 1 TFs both clustered together with the PIT1 tumors. To further explore the transcriptome-based clustering we performed a Principal Component Analysis (PCA) to demonstrate the relation between the three TF classes based on the global gene expression data. The data show that the SF1 and PIT1 tumors appear more homogenous and form more tight clusters as compared to the more heterogeneous group of TPIT tumors (Fig. 1b).

### Expression patterns of transcription factors and pituitary hormones

**Fig. 2:**
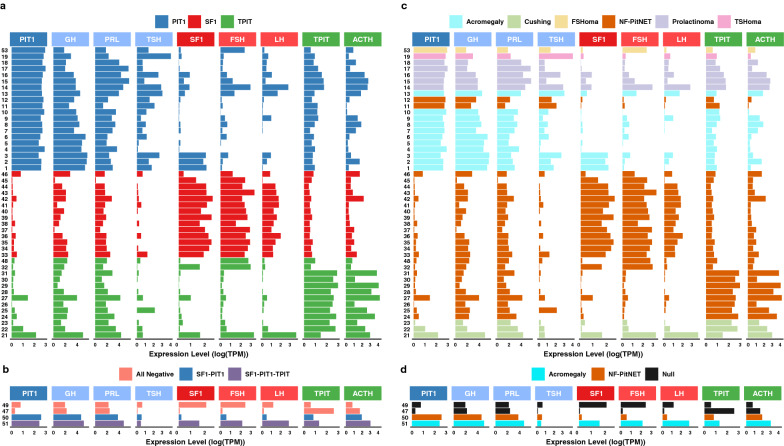
Expression patterns of transcription factors (SF1, PIT1 and TPIT) and the six pituitary hormones (GH, PRL, TSH, LH, FSH and ACTH) across the analyzed PitNETs. **a** Samples are colored according to their transcription factor classes. **b** Four samples are colored according to their transcription factor expressions. **c** Samples are colored according to their functional status classes. **d** Four samples are colored according to their functional status or null class

Next, we analyzed the expression pattern of the three TFs and corresponding pituitary hormones. As expected, the results show that the expression levels of TFs and the corresponding hormones in general follow the current classification of PitNETs with highest expression of PIT1 together with GH, PRL and TSH in the PIT1 class, SF1 together with FSH and LH in the SF1 class, and TPIT together with ACTH in the TPIT class. Overall, there is a clear consistency in high expression level of a TF and the corresponding hormone(s) (Fig. 2a). It is also evident that the expression levels of TFs and hormones is heterogeneous with individual tumors showing divergent unexpected expression levels of certain TFs or hormones, e.g., PIT1 tumors with relative high levels of TPIT/ACTH, TPIT tumors with relative high expression of PIT1/GH and PRL. Interestingly, three PIT1 tumors, all categorized as densely granulated somatotroph tumors, also show relatively high levels of SF1 expression but without increased levels of corresponding FSH and LH, whereas a single functioning FSH-oma demonstrates high PIT1 and FSH expression in accordance with the immunohistochemical results (Fig. 2a). Two of the 51 PitNETs were classified as expressing two (PIT1 + SF1) or all three TFs, and two PitNETs were classified as “null cell tumors” since they were negative for TFs and adenohypophysial hormones in the IHC-based classification. The expression levels of TFs and corresponding hormones for these four uncommon PitNETs are displayed in Fig. 2b. In two cases of tumors expressing more than one TF, the gene expression profile corresponds to the IHC profile in the triple PitNET, whereas the results are disparate in the double PIT1 + SF1 tumor. The expression levels of TFs and hormones in the two “null cell tumors” appear to some extent inconsistent with IHC results, with one negative tumor showing relatively high expression levels of SF1/FSH and one with high expression levels of TPIT/ACTH (Fig. 2b). Expression levels of TFs and hormones for all tumors are shown in Additional file 2: Table S4.

### Comparison of non-functioning and hormone producing tumors

To explore if expression levels of the main TFs and corresponding hormones differed between non-functioning tumors and hormone producing/secreting tumors a similar analysis was performed. The results, summarized in Fig. 2c, d, show that both TFs and hormones are expressed at essentially the same levels independent of functional state, indicating that the difference between non-functioning and functioning PitNETs is not directly linked to expression patterns of these central TFs and pituitary hormones. Next, we aimed to investigate the differences in global gene expression patterns between functioning and non-functioning PitNETs. We found that 26 genes showed significant differential expression (adjusted *p *value < 0.05 with absolute log fold change > 2). The limited list of genes differentially expressed in non-functioning compared to functioning PitNETs included both pituitary transcription factors and hormones as well as other known genes (Additional file 1: Figure S1a), with expression levels across the PitNET cohort for the top 10 differentially expressed genes shown in Additional file 1: Figure S1b. The full results are presented in Additional file 2: Table S5. Although functioning and non-functioning PitNETs appear separated clusters in a PCA analysis as well (Additional file 1: Figure S1c), the analysis of differential expression showed no significant differential expression when adjusting for the effect of PIT1, SF1 and TPIT, suggesting that the observed clustering is mainly driven by the three TF-related subgroups.

### Correlation network analysis

**Fig. 3:**
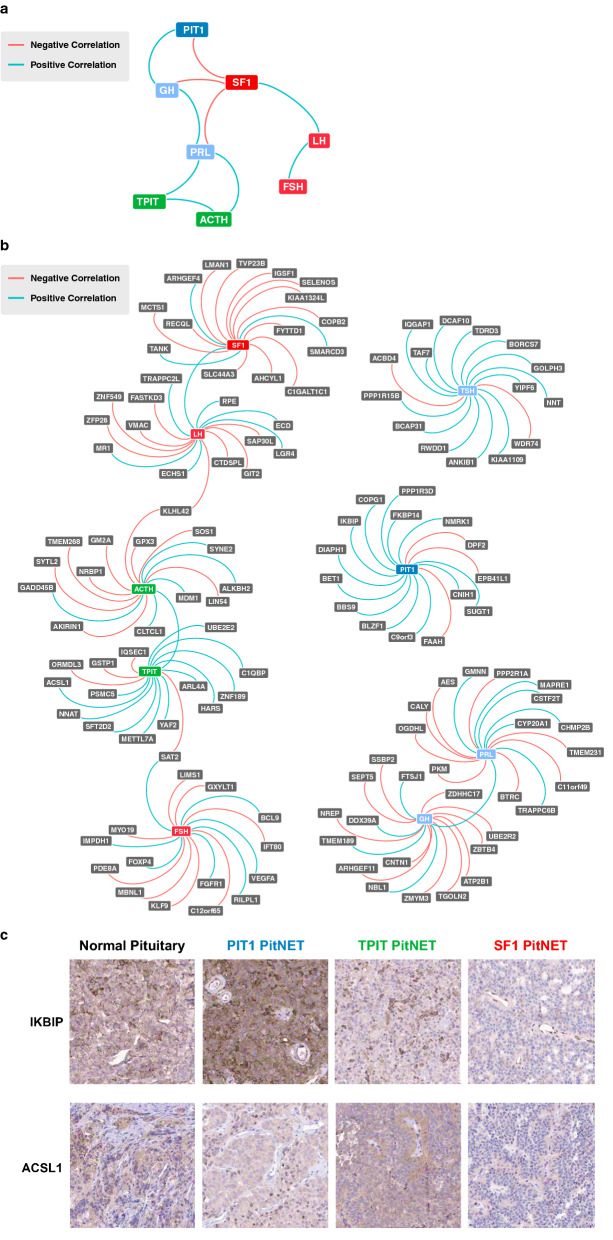
Correlation network analysis. **a** A network visualization showing the Spearman correlation between transcription factors (SF1, PIT1 and TPIT) and the six pituitary hormones (GH, PRL, TSH, LH, FSH and ACTH). **b** A network visualization showing the top fifteen neighbors based on Spearman correlation connected to transcription factors (SF1, PIT1 and TPIT) and six pituitary hormones (GH, PRL, TSH, LH, FSH and ACTH). **c** Immunohistochemistry using antibodies recognizing IKBIP (top) and ACSL1 (bottom) in three different PitNETs representing the three TF classes of PitNETs. IKBIP, correlating with PIT1, shows strong positivity in PIT1 tumor cells whereas tumor cells in TPIT and SF1 tumors are essentially negative. ACSL1, correlating with TPIT, shows positivity in a TPIT tumor whereas PIT1 and SF1 tumors appear negative or only faintly positive. Magnification corresponds to 200 × for all the microphotographs

Next, we performed a network analysis to explore correlations (Spearman) between the main TFs and pituitary hormones. The results showed that there were 10 significant correlations (Fig. 3a). As expected, the expression levels of PIT1 showed a positive correlation with expression levels of GH, TPIT with ACTH, and SF1 with LH. There was no significant correlation between PIT1 and PRL or TSH, and no significant correlation between SF1 and FSH. FSH and LH correlated, as expected for the hormones belonging to the same cell lineage. Interestingly, there was a significant correlation between TPIT and PRL and an inverse correlation between SF1 and PIT1, GH and PRL (Fig. 3a). The results indicate that expression of a pituitary TF and the corresponding hormone(s) are not independent networks, but rather that there is a crosstalk between the three cell lineages in PitNETs. To compare with normal pituitary gland, we explored the correlations between TFs and hormones in publicly available RNA-Seq data from GTEx (n = 183) [9]. The correlations in normal pituitary gland show a more expected result without crosstalk between PIT1, SF1 and TPIT cell lineages (Additional file 1: Figure S2). To further explore genes with significant correlations to the pituitary TF and hormone expression levels, a Spearman correlation analysis was performed. The top 15 neighbor correlated genes, independent if positive or negative correlation, are shown in Fig. 3b. A majority of the top correlated genes have essentially unknown functions and role in endocrine biology. The correlation analysis only showed few expected connections between sub-network modules, i.e. SF1 and LH, TPIT and ACTH, and PRL and GH. Both *SAT2*, bridging FSH and TPIT networks by showing positive correlation to FSH and negative correlation to TPIT, and *KLHL2*, bridging LH and ACTH networks by showing negative correlation with both LH and ACTH, have not been previously implicated in pituitary functions. Only few well-known genes were among top 15 genes that correlate with TFs, including *IGSF1*, with a pituitary gland specific expression pattern, and *NNAT* (neuronatin), with enriched expression in brain, pituitary gland and placenta, both known to be involved in the development and maturation of pituitary gland. *FADH*, *ASCL1*, *C1QBP* and *GSTP1*, involved in metabolic and signaling processes are also previously known genes that showed strong correlations to expression patterns of TFs. To further explore a few previously unknown genes that showed strong correlation to TFs, we selected *IKBIP* correlating with PIT1 and *ACSL1* correlating with TPIT to validate if also corresponding proteins showed differences in expression patterns between different PitNETs. *IKBIP*, a novel marker of epithelial-mesenchymal transition (EMT) predicting poor prognosis in gliomas [54], was found to be expressed in blood vessels of all PitNETs and showed strong cytoplasmic positivity in PIT1 tumor cells and was essentially negative in tumor cells from SF1 and TPIT tumors (Fig. 3c). The *ACSL1* encoded protein, involved in fatty acid metabolism, was positive in tumor cells from TPIT tumors and only very weak or negative in tumors of the PIT1 and SF1 lineage (Fig. 3c). There were also relatively few well-known genes correlating with expression patterns of adenohypophysial hormones. A positive correlation to FSH was found for *FGFR1* and *VEGFA*, well-known genes involved in pituitary hormone regulation and angiogenesis. In contrast, *KLF9* suggested to be involved in the regulation of the pituitary-thyroid axis and *PDE8A* involved in testosterone synthesis showed negative correlation to FSH. A positive correlation to LH was found for *LGR4* that regulates expression of estrogen receptor and *MR1* that mediates tumor immune escape, whereas there was a negative correlation between LH and *GIT2*, implicated in aging and cellular senescence. *GADD45B*, involved in pituitary growth and tumorigenesis showed a positive correlation to ACTH, whereas *GPX3*, involved in the protection from oxidative damage, and *SOS1* and *SYTL2*, both involved in signaling and secretory pathways, showed a negative correlation to ACTH gene expression. TSH expression correlates to *NNT* involved in mitochondrial metabolism and glucocorticoid deficiency, and *BCAP31* involved in signaling processes. *AES*, the binding factor to *PROP1*, a marker of PIT1 cell lineage progenitor cells, correlated negatively to PRL expression. Genes correlating positively to GH have not been previously reported in relation to the pituitary gland, except PRL. Sparse data indicate potential hypothalamo-pituitary involvement of *CNTN1* and *NREP*, two genes that correlate negatively to PRL. Correlation coefficients for TFs, hormones and co-expressed genes are listed in Additional file 2: Table S6.

### Differential expression analysis

**Fig. 4:**
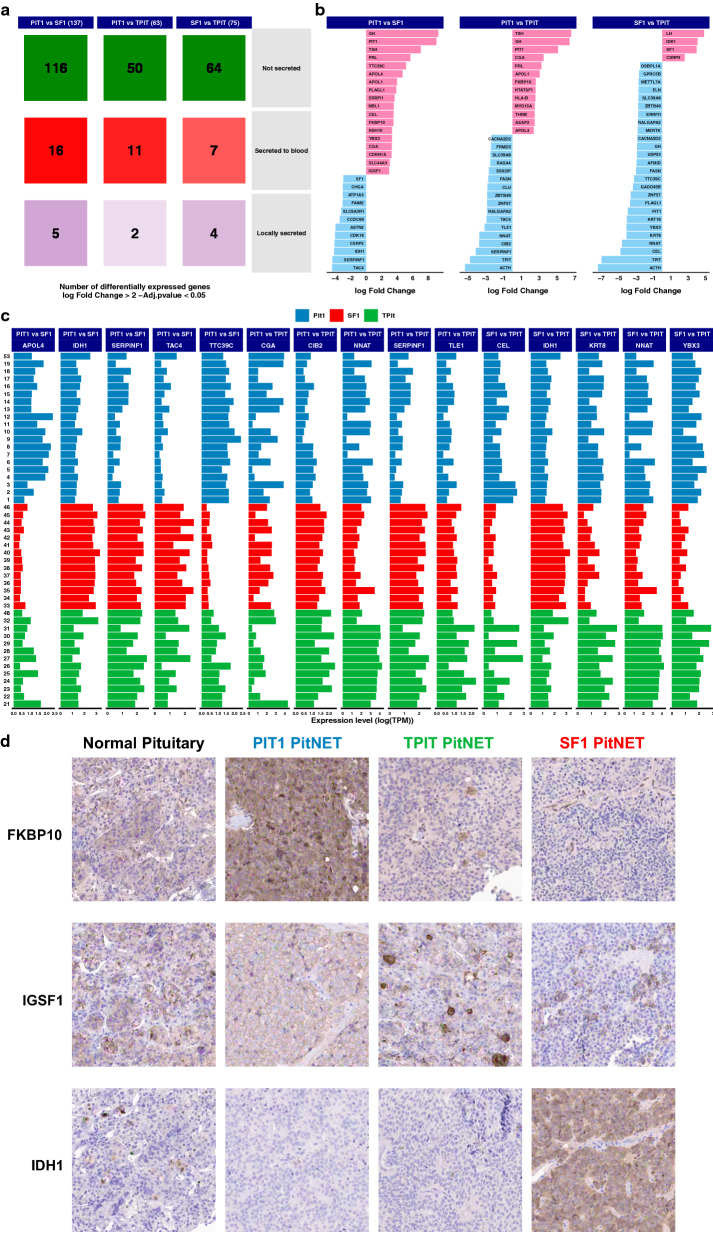
Differential expression analysis between the transcription factor related groups. **a** Overview of the differentially expressed genes and their secretome class according the Human Protein Atlas (www.proteinatlas.org). **b** Top 30 differentially expressed genes sorted according to their respective log fold change. **c** Barplot of the expression levels of the top five differentially expressed genes in each TF-related comparison. **d** Immunohistochemistry examples of differentially expressed proteins between TF classes of PitNETs. FKBP10 (top) shows a distinct cytoplasmic positivity in a PIT1 tumor as compared to TPIT and SF1. IGSF1 (middle) shows a clear and general membraneous positivity in PIT1 tumor cells, whereas only few cells appear positive in a TPIT and a SF1 tumor. IDH1 (bottom) shows a distinct cytoplasmic positivity in a SF1 tumor compared to negative staining in a TPIT and a PIT tumor. Magnification corresponds to 200 × for all the microphotographs

To further characterize the difference between the TF classes of tumors, we next performed a differential expression analysis excluding genes with very low expression levels. Altogether 275 genes were significantly differentially expressed (adjusted p value < 0.05) in the three comparisons with an absolute log fold change > 2 (Additional file 2: Table S7), with highest number of differentially expressed genes (137) found between PIT1 and SF1 tumors. The majority of differentially expressed genes encode non-secreted proteins, but several genes encoding proteins secreted to blood were also differentially expressed (Fig. 4a). The top 30 differentially expressed genes in the three analyses are shown in Fig. 4b. Expression levels across all tumors are also shown for the top five differentially expressed genes in each comparison (Fig. 4c). The analysis shows as expected that several of the TFs and pituitary hormones are among the top differentially expressed genes in the group comparisons. However, many of the genes differentially expressed between SF1, TPIT and PIT1 group were genes poorly described or with unknown functions in pituitary biology. However, many of the genes differentially expressed between SF1, TPIT and PIT1 group were genes poorly described or with unknown functions in pituitary biology. Interestingly, most of these genes have been implicated in general mechanisms of tumor biology, such as *SERPINF1*, reported as an inhibitor of angiogenesis and tumor suppressor [58], *ASAP2*, an EGFR pathway activator [15], or *ELN* and *KRT8* related to the process of EMT [22, 52]. Some of the genes have also been associated with development of specific tumors, e.g. TAC4 [5], *IGSF1* [17] and *SLC39A8* (ZIP8) [24]. A prognostic role has also been reported for some of these genes, both negative (*ERRFI1*) [25], and positive (*SLC44A3*) [29]. Additionally, several of the differentially expressed genes are proposed to be potential therapeutic targets, such as *FAIM2* in small cell lung carcinoma [18], *ERRFI1* in pancreatic carcinoma [25], *RASA4* in triple-negative breast carcinoma [53], *FKBP10* in gliomas [7, 18], metastatic gastric carcinoma [16] and lung carcinoma [38], and *CACNA2D* in prostate carcinoma [18]. Overview of the most interesting genes differentially expressed between the cell-lineage based groups of PitNETs has been presented in Table 2. Concordance between gene and protein expression for a few selected genes, *FKBP10*, significantly overexpressed in PIT1 tumors compared to SF1 and TPIT tumors, *IDH1*, up-regulated in SF1 tumors as compared to both PIT1 (> 15-fold) and TPIT (nearly fivefold), and *IGSF1* showing high expression in PIT1 tumors as compared to both SF1 and TPIT tumors, was demonstrated using immunohistochemistry (Fig. 4d).

**Table 2:** Overview of the selected differentially expressed genes between the cell-lineage based groups of PitNETs

Gene	Function	Differential expression
*ZBTB49*	Tumor suppressor gene	TPIT versus PIT1 TPIT versus SF1
*RALGAPA2*	Tumor suppressor gene	TPIT versus PIT1 TPIT versus SF1
*NNAT*	Regulation of ion channels during development of pituitary gland and brain; Enriched expression pattern in adult pituitary gland, brain and placenta	TPIT versus PIT1 TPIT versus SF1
*CACNA2D*	Potentially druggable cancer-related gene in prostate carcinoma [55]	TPIT versus PIT1 TPIT versus SF1
*SLC39A8 (ZIP8)*	Both an anti-oncogenic and pro-oncogenic role; downregulated in breast cancer cells compared to the normal tissue [24]; upregulated in neuroblastoma cells [33]	TPIT versus PIT1 TPIT versus SF1
*ELN*	Tumor promoting gene, related to the process of EMT [22, 52]	TPIT versus PIT1 TPIT versus SF1
*KRT8*	Tumor promoting gene, related to the process of EMT [22, 52]	TPIT versus PIT1 versus SF1
*RASA4*	Increased proliferation and apoptosis resistance, a potential therapeutic target in triple-negative breast cancer [54]	TPIT versus PIT1
FKBP10*	Overexpression in gliomas [7], metastatic gastric carcinoma [16] and lung carcinoma; potential therapeutic target	PIT1 versus SF1 PIT1 versus TPIT
*SLC44A3*	Potential protective role; prognostic marker in uveal melanoma [29]	PIT1 versus SF1 PIT1versus TPIT
*ASAP2*	EGFR signaling pathway activator; can be targeted by an antiparasitic drug [15]	PIT1 versus TPIT
IGSF1*	PIT1 cells differentiation and hormone regulation; cell proliferation and apoptosis in thyroid cancer [17]	PIT1 versus SF1
*ERRFI1*	Negative prognostic factor; potential therapeutic target in pancreatic carcinoma [25]	TPIT versus SF1 PIT1 versus SF1
*USP53*	Tumor suppressor gene	TPIT versus SF1 PIT1 versus SF1
*CIB2*	Tumor suppressor gene	TPIT versus PIT1 SF1 versus PIT1
*SERPINF1*	Enhanced expression in retina; strong inhibitor of angiogenesis; neurotrophic protein; tumor suppressor gene	TPIT versus PIT1 SF1 versus PIT1
*TAC4*	Promoter of cell migration in gliomas, melanomas and breast adenocarcinomas [5]	TPIT versus PIT1 SF1 versus PIT1
*FAIM2*	Potential neuroendocrine marker; potential therapeutic target in small cell lung carcinoma [18]	SF1 versus PIT1
IDH1*	A mitochondrial enzyme; well-known cancer associated gene	SF1 versus. PIT1 SF1 versus TPIT

*Concordance between protein and gene expression demonstrated using IHC as shown in Fig. 4d

To further explore more general differences in gene expression between the three different TF classes, we analyzed the genes with highest expression levels in PitNETs. As for human tissues in general, the genes with highest expression levels were dominated by mitochondrially encoded enzymes involved in the mitochondrial electron transport chain. Twelve of the top 15 genes across all PitNETs were mitochondrially encoded enzymes and the three other genes encoded for two of the pituitary hormones GH and PRL, and the G protein *GNAS*, mutated in up to 50% of sporadic somatotroph tumors [14, 20, 41]. Interestingly, the SF1 tumors show a substantially higher expression level of mitochondrially encoded enzymes compared to PIT1 and TPIT tumors suggesting that SF1 PitNETs are metabolically different with a relatively high mitochondrial (cell respiratory chain) activity. Top 50 expressed genes are shown in Additional file 2: Table S8.

We have also explored the overlap of the abovementioned differentially expressed genes with those related to gender, invasiveness and tumor size (Additional file 2: Table S9 and S10). No overlap has been observed with invasiveness-related genes. Very few genes showed overlap with gender and include: *ELN, MERTK, UCK2, AFMID.* However, tumor size showed different overlaps; six genes overlap with PIT1 vs SF1 (*YBX3, CGA, ENPP2, BAG3, KRT8, SULF2*), five genes overlap with PIT1 vs TPIT (*CACNA2D2, CGA, KRT8, RALGAPA2, PDE7A*), and eight genes overlap with SF1 vs TPIT (*CACNA2D2, BRCA1, YBX3, NFIL3, KRT8, RALGAPA2, SULF2, PDE7A*).

## Discussion

Here, we have analyzed the genome-wide transcriptomes from a well-characterized cohort of 51 PitNETs, to identify gene expression patterns and to compare how global gene expression signatures correlate to the current classification of PitNETs based on immunohistochemistry. The overall results support the 2017 WHO classification and show that PitNETs can be divided into three different tumor classes and that these three tumor classes reflect the main expressed pituitary specific transcription factors and corresponding adenohypophysial hormone(s) as identified by immunohistochemistry. However, the expression levels of TF genes appear more heterogeneous within the PitNET groups as compared to the corresponding normal cell lineages in the pituitary gland, suggesting an interplay between pituitary cell-lineages during the PitNET development. Novel genes with possible role in PitNET development have emerged, several of those being potentially targetable.

The TF classes of PitNETs appear as clearly separated clusters with distinct gene expression profiles. Although there is a clear predominance of the expected TF and hormone(s) within each tumor type, it is evident that expression levels of these genes are heterogeneous with variable expression levels of most TFs and hormone(s) in almost all tumors. It cannot be ruled out that part of variability could be due to admixture of normal pituitary epithelial cells, however, the high fraction of tumor cells (80–90%) for almost all tumors suggests that variability is more likely a consequence of variable expression levels of these genes in the tumor cell population. Two other recently published studies also demonstrated transcriptomics results that generally support the cell-lineage based classification of PitNETs [35, 47].

The PIT1 tumors appear the most distinct with respect to expression levels of TF although individual tumors also show relatively high expression levels of SF1 and unrelated hormone(s) such as FSH, LH and ACTH. Interestingly, three PIT1 tumors that show relatively high levels of SF1 but without increased levels of corresponding FSH and LH represent the three densely granulated somatotroph tumors included in our cohort. In a study by Neou et al. [35], SF1 was at high level in *GNAS* wild type somatotroph tumors. Although mutational analysis of *GNAS* was not performed, increased SF1 expression in densely granulated somatotroph tumors is an interesting finding having in mind that there is no explanation yet for biological and clinical differences between sparsely and densely granulated somatotroph tumors. Moreover, a single clinically functioning gonadotroph tumor in our cohort demonstrates, in addition to FSH, also high PIT1 expression, which is in accordance with the immunohistochemical results in this peculiar tumor. A phenomenon of the overlapping SF1 and PIT1 gene signatures, which can rarely be observed also at the protein level in uncommon true plurihormonal PitNETs, requires further studies as it may have impact on functionality of the tumors belonging to the two cell lineages and bring us closer to an explanation of silencing of gonadotroph tumors. The TPIT tumors also include a few cases with relatively high expression of unrelated FSH and/or LH. However, it does not seem to be related to a functional state of corticotroph tumor as suggested in a previous study where overlap with gonadotroph signature was found in silent corticotroph tumors [35]. SF1 tumors appear more homogenous with low expression levels of PIT1, TPIT, and unrelated hormones.

In the analyzed cohort, there were a few rare tumors including two cases with negative immunohistochemistry designated as “null cell adenomas”. One of these showed high expression of SF1 and corresponding hormones and the other showed relatively high expression of TPIT with more moderate expression levels of ACTH. Interestingly, these two “null cell adenomas” clustered with the expected respective TF-class suggesting that these tumors are not distinct outliers, consistent with results in a recent study by Neou et al.[35]. This further supports the speculation that so called “null cell adenomas” can represent silent gonadotroph (SF1) or corticotroph (TPIT) PitNETs that could not be correctly classified due to potential preanalytical or analytical problems related to immunohistochemistry [30, 31]. It cannot be ruled out that, in these specific tumors, the discrepancy between the RNA transcript and protein expression is due to posttranslational processes, mutations or truncated transcripts that indeed affect the level of protein content or its antigen characteristics. Two additional rare cases included one double PitNET with both a PIT1 and SF1 component and one triple PitNET with all three TF components in immunohistochemistry. Both tumors were clustered in the PIT1 cluster, and also showed predominant high levels of PIT1 transcripts and corresponding hormone(s) although lower levels of the other TFs and hormone(s) were also expressed. A possible explanation for this could be a predominance of the PIT1 component in the frozen tumor tissue used for RNA-Seq.

Our analyses showed that the global gene expression patterns reflected well three distinct classes of tumors signified by the expression pattern of the main pituitary TFs, i.e., SF1, PIT1 and TPIT. The analyzes of differentially expressed genes that distinguish the different SF1, PIT1 and TPIT driven tumors showed expected differential expression of the pituitary TFs and hormone(s) including CGA that encodes common alpha-subunit of glycoprotein hormones TSH, FSH, LH and choriogonadotropin, but also included some previously well-known genes implicated in pituitary biology and PitNETs.

*PLAGL1* (a zinc finger protein, ZAC1), a tumor suppressor gene [49, 51], a senescence marker p21 (*CDKN1A*) [32], and *IGSF1* (Immunoglobulin superfamily member 1), a plasma membrane glycoprotein that seems to be a coreceptor in inhibin signaling [13], all with known roles in pituitary tumorigenesis, were upregulated in PIT1 positive tumors. TPIT tumors overexpressed neuronantin, which is silenced by promotor hypermethylation in PitNETs [39] and also *MERTK*, a receptor tyrosine kinase overexpressed in a variety of neoplasm and a potential therapeutic target [10]. *GADD45B*, a stress response gene and a tumor suppressor [59] was significantly downregulated in SF1 tumors.

Although not among the top differentially expressed genes, *TGFBR3L*, recently reported as a marker of gonadotroph cell differentiation [44], appears upregulated in gonadotroph tumors confirming our previous results at the protein level. Moreover, *TGFBR3L* showed a relative high expression level in the triple PitNET containing a gonadotroph component, in one “null cell adenoma” that showed a gonadotroph mRNA profile, and in the PIT1 positive FSH-oma, all well consistent with gonadotroph expression of *TGFBR3L*. Interestingly, a relatively high expression level was also evident in one PIT1 and one TPIT tumor, both with high mRNA expression of gonadotroph markers, supporting an interplay between the pituitary cell lineages in PitNET development.

Our findings show that SF1 tumors appear to express relatively high levels of the mitochondrially encoded genes suggesting a high mitochondrial (cell respiratory chain) activity due to high cell energy expenditure. One such differentially expressed gene is isocitrate dehydrogenase (*IDH*) [11]. Inactivating *IDH* gene mutations lead to reduced NADPH production and accumulation of hypoxia-inducible factor 1 that activates signaling pathways important for growth of adult low-grade gliomas [57]. Except an immunohistochemical study that showed lack of the frequent *IDH1*(R132H) variant in PitNETs [8], *IDH* has not been studied more closely in PitNETs. However, in a previous proteomic study [55], differentially expressed proteins related to mitochondrial metabolism were described between non-functioning gonadotroph tumors and normal pituitary gland. Moreover, Neou et al. recently reported high oxidative phosphorylation in gonadotroph tumors in the transcriptomic analysis [35]. As gonadotroph (SF1) tumors are non-functioning in almost all cases, one can speculate whether mitochondrial processes may play a role in silencing of these tumors. We have looked closer at the differences in global gene expression between functioning and non-functioning PitNETs. Although the two groups cluster separately, we could not identify significant differences in gene expression beyond clustering according to TFs.

A large fraction of the genes differentially expressed between SF1, TPIT and PIT1 group were genes poorly described or with unknown functions in pituitary biology. Interestingly, most of these genes have been associated with malignancies in different tissues and organs, either as tumor promoting genes (*ERRFI1, FKBP10, TAC4, ASAP2, CACNA2D, RASA4, SSX2IP, ELN, KRT8*), tumor suppressors (*SLC44A3, SERPINF1, ZBTB49, RALGAPA2, CIB2, USP53*), or both (*SLC39A8*). Upregulation of the tumor suppressor genes in pituitary tumors is an interesting phenomenon, that may, at least in part, explain usually benign clinical course of PitNETs. Furthermore, many of the genes that we found upregulated in PitNETs have been reported as potential therapeutic targets, e.g. *FAIM2* [18], *ERRFI1* [25], *RASA4* [53], *FKBP10* [7, 18], and *CACNA2D* [18]. This is of interest mostly for patients with Cushing disease, NF-PitNETs and patients with aggressive PitNETs, who presently can be offered none or only limited pharmacological therapeutic options.

A few differentially expressed genes between the PitNET subtypes also correlate with tumor size (*CACNA2D2, CGA, KRT8, RALGAPA2*) suggesting a potential role in tumor growth.

Although some of differentially expressed genes are identical across our and the two other recently reported studies [35, 47], each study reveals also novel genes with potential tumorigenic and therapeutic implications, indicating a need to study gene expression profile in different PitNET cohorts. The only partial overlap is expected due to differences in criteria for classification, technology platforms, pipelines, cut-offs etc., emphasizing the need for further studies of gene expression profiles in different well-characterized PitNET cohorts.

For selected differentially expressed genes (*IKBIP, ACSL1, FKBP10, IGSF1*, and *IDH1*), a gene expression pattern was also explored at the protein level by using immunohistochemistry, showing a high concordance between mRNA and protein expression, giving additional strength to our transcriptomics-based data.

A relatively low number of functioning corticotroph tumors included is a limitation of the study. Functioning corticotroph tumors are, however, rare, and usually microtumors with sparse amount of representative tumor tissue in surgical specimens, which presents a limitation factor for tissue-based studies.

In conclusion, transcriptomics data, in our well-characterised cohort of PitNETs of different types, give an overall support to the current pituitary cell lineage-based classification of pituitary tumors, with a certain gene signature overlap reflecting an interplay between different cell-lineages in the development of PitNETs. Although based on only two cases, our results support the hypothesis that the so called “null cell adenomas” do not represent a distinct entity, but rather misdiagnosed silent gonadotroph or corticotroph tumors. Novel genes, previously unknown in pituitary physiology, have emerged and their role as PitNETs’ drivers and potential therapeutic targets should be explored in future studies.

## Supplementary Information

**Additional file 1.** Supplementary figures 1 and 2. Figure S1: Differential expression analysis between functioning (F-PitNET) and non-functioning (NF-PitNET) tumors; Figure S2: Correlation network analysis in normal pituitary gland.

**Additional file 2.** Supplementary tables 1-10 (S1-10). Table S1: Background PitNET cohort; Table S2: Antibodies used for IHC; Table S3: Expression levels (TPM) of all genes in all cases; Table S4: Expression levels of pituitary transcription factors and hormones accross all cases; Table S5: Differentially expressed genes between non-functioning (NF-PitNETs) and functioning PitNETs (F-PitNETs); Table S6: Genes with significant correlation to pituitary transcription factors and hormones; Table S7: Differentially expressed genes between TF classes of PitNETs; Table S8: Expression levels in TF classes of the 50 genes with highest expression levels in PitNETs; Table S9: Significantly correlated genes with tumor size; Table S10: Differentially expressed genes related to gender or invasiveness. 

## Data Availability

The datasets used and/or analyzed during the current study are available from the corresponding author on reasonable request.

## Abbreviations

PitNET: Pituitary neuroendocrine tumor
TF: Transcription factor
IHC: Immunohistochemistry
SF1: Steroidogenic factor-1
PIT1: Pituitary transcription factor 1
TPIT: T-box family member 19 (TBX19)
NF: Non-functioning
F: Functioning
DG: Densely granulated
SG: Sparsely granulated
NG: No granulation
FSH: Folliculostimulating hormone
GH: Growth hormone
PRL: Prolactin
TSH: Thyroid stimulating hormone
ACTH: Adrenocorticotrophic hormone
