# Supplementary material for: Annotation of pituitary neuroendocrine tumors with genome-wide expression analysis
Source: Acta Neuropathol Commun. 2021 Nov 10;9:181. doi: 10.1186/s40478-021-01284-6 (PMC8579660; doi:10.1186/s40478-021-01284-6)
Supplement: Supplementary file 1 — Additional file 1. Supplementary figures 1 and 2. Figure S1: Differential expression analysis between functioning (F-PitNET) and non-functioning (NF-PitNET) tumors; Figure S2: Correlation network analysis in normal pituitary gland. [file 40478_2021_1284_MOESM1_ESM.docx]

**
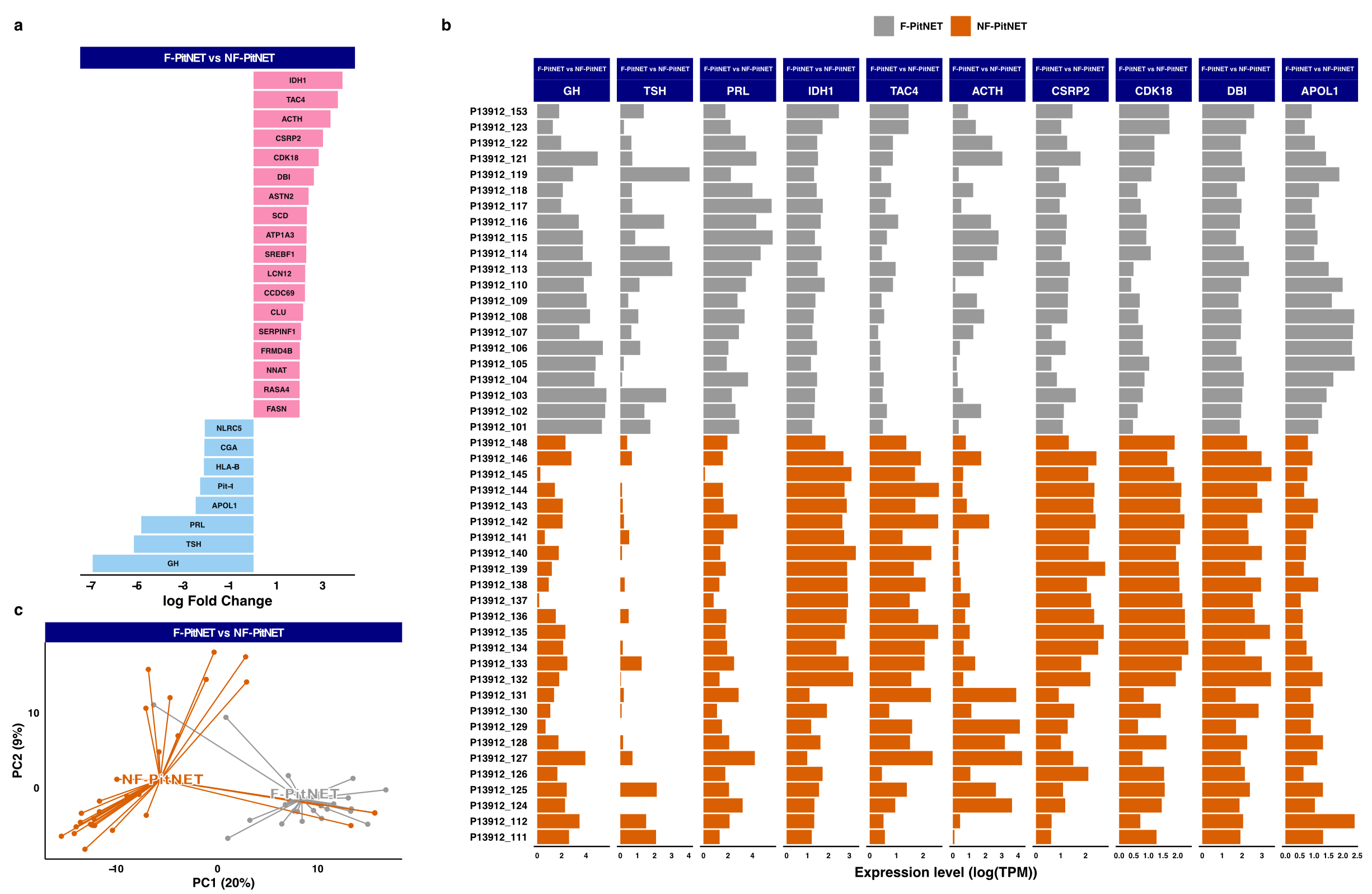
**

**Supplementary Figure 1.** Differential expression analysis between Functioning (F-PitNET) and Non-functioning (NF-PitNET) tumors. **a** Overview of the 26 significantly differentially expressed genes. **b** Expression pattern of the top fifteen genes between F-PitNET and NF-PitNET across the analyzed tissues. **c** Principal component analysis showing the relationship between all the analyzed tissue colored according to their respective functional status classes. (F-PitNET: Functioning Pituitary Neuroendocrine Tumors, NF-PitNET: Non-Functioning Pituitary Neuroendocrine Tumors).


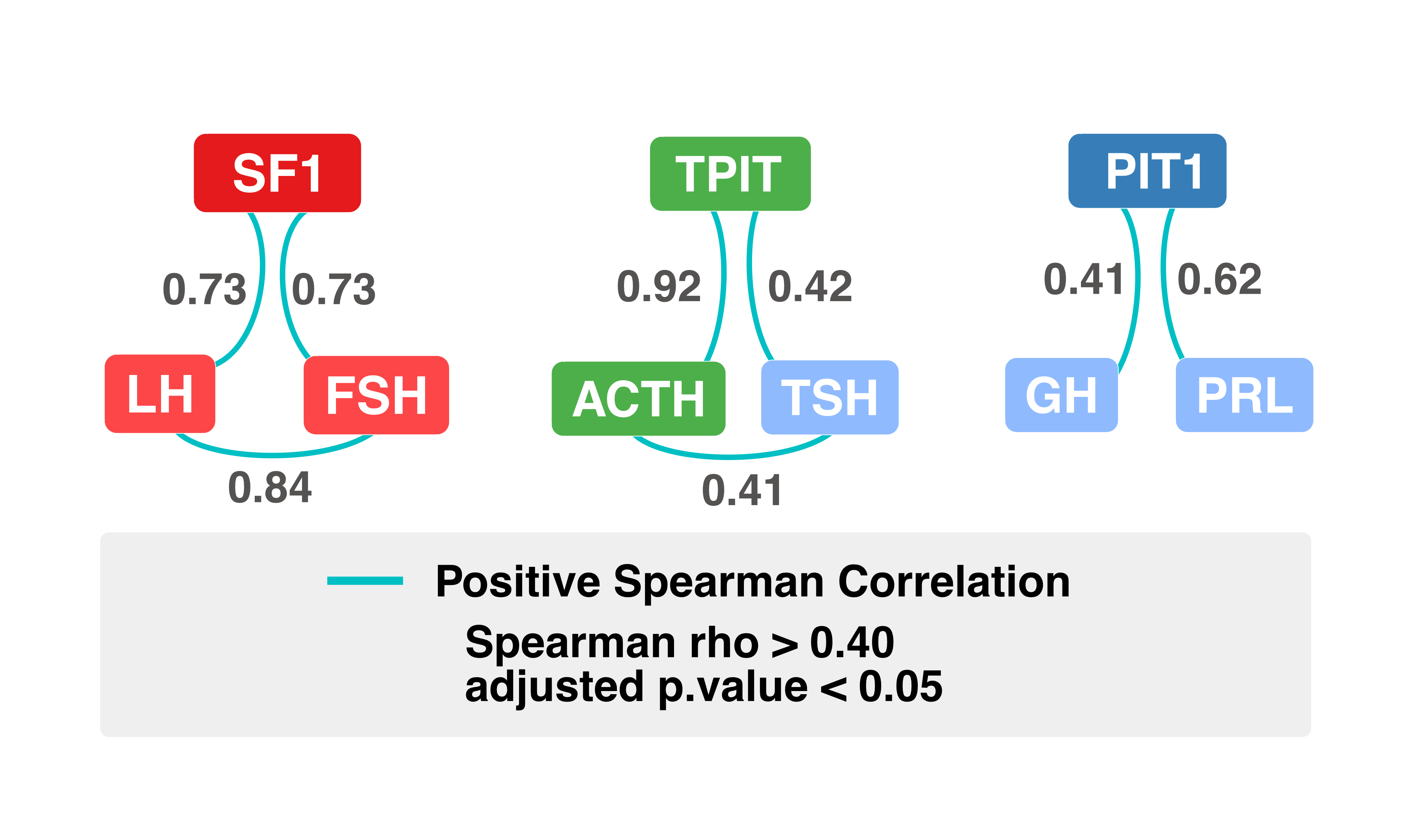


**Supplementary Figure 2.** Correlation network analysis in normal pituitary gland. A network visualization showing the Spearman correlation between transcription factors (SF1, PIT1 and TPIT) and the six pituitary hormones (GH, PRL, TSH, LH, FSH and ACTH). Based on data from 183 specimens from normal human pituitary gland (RNA-Seq data from GTEx)
